# A Hierarchical Solution Approach for Occupational Health and Safety Inspectors' Task Assignment Problem

**DOI:** 10.1016/j.shaw.2021.01.004

**Published:** 2021-01-26

**Authors:** Feyzan Arikan, Songul K. Sozen

**Affiliations:** 1 Faculty of Engineering, Department of Industrial Engineering, Gazi University, 06570 Maltepe, Ankara, Turkey; 2 Ministry of Labour and Social Security, Labour Inspection Board, 06520 Ankara, Turkey

**Keywords:** Legislation and regulations, Mathematical modeling, Non-technical human skills, Organizational aspects, Safety management and policy

## Abstract

**Background:**

Occupational health and safety (OHS) is a significant interest of all governments to prevent workplace hazards. Although appropriate legislation and regulations are essentials for the protection of workers, they are solely not enough. Application of them in practice should be secured by an efficient inspection system. Fundamental components of an inspection system are inspectors and their audit tasks. Maintaining the fair balanced task assignment among inspectors strictly enhances the efficiency of the overall system.

**Methods:**

This study proposes a two-phased goal programming approach for OHS inspectors' task assignments and presents a case study.

**Results:**

The solution approach gives the balanced assignment of inspectors to the workplaces in different cities of the country in the planning period. The obtained schedule takes into account the distances covered by the work places and the number of the workplaces' employees to be audited and pays attention to the human factors by considering the preferences of the inspectors. The comparisons between the obtained optimal schedule and the implemented one that is produced manually show that the approach not only maintains the technical requirements of the problem, but also provides social and physical balance to the task assignment.

**Conclusion:**

Both the approach and the application study are expected to offer fruitful inspirations in the area of safety management and policy and they provide a good guide for social policy and organizational aspects in the field of OHS inspectors' task assignment.

## 1 Introduction

Healthy workers and appropriate working conditions not only ensure enhancing the quality of life of individuals and society but also contribute to productivity of workplaces and socioeconomic development of countries. To reach these benefits, occupational health and safety (OHS) is the key concept for all governments. OHS is a dynamic process and the objectives are long term. Improvements in a long-term objective project can be obtained with a strict control on the implementation phases. Hence, OHS policy should be supported by legislation and regulations, along with an adequate inspection system to ensure that these are enforced. The labor inspectors are the executive agents of inspection systems and carry out the actual work of enforcement [1].

Labor inspectors' roles and responsibilities are determined by regulations. They are mainly entrusted to measure, audit, and evaluate the effectiveness of hazard controls and hazard control programs, to execute enforcement tools and to write reports. Inspectors are responsible to respect the principles of honesty, independence, impartiality, reliability, and competence. They should evoke respect and dignity in their behaviors and actions. They are obliged not to disrupt, stop and complicate the normal course of work and the operation of the workplace as much as possible depending on the nature of the subject they are studying.

The fulfillment of inspectors' roles and responsibilities are directly related with nontechnical human skills constituting situation awareness, decision making, communication, team working leadership, managing stress, coping with fatigue [2], and highly depends on work engagement [3], which includes a positive, affective-motivational state of work-related well-being that is characterized by vigor, dedication, and absorption [4]. The key concept to ensuring work engagement is fairness or “justice” based on the social psychology literature [5].

In organizational psychology, theories of fairness or “justice” have been classified under four headings [6]: The fairness in distribution of resources is called Distributive Justice [7]. The fairness in decision making process is termed as Procedural Justice [8]. The fairness in interpersonal treatment is known as Relational (or Interactional) Justice [9]. Collectively, Distributive Justice, Procedural Justice, and Relational Justice compose Organizational Justice [10]. The term Organizational Justice was first coined by Greenberg [11]. It refers to people's perception about organization's fairness and its reactions toward such perception. Unfair treatment not only decreases job performance but also reduces quality of work and degree of cooperation among workers [12].

The relationship between organizational justice [13] and worker productivity [14] has all been the widest and longest research tradition [6]. The organizational justice has also direct effects on work engagement [15] turnover and job satisfaction [16].

Incorporation of fairness in inspectors' task assignment results job satisfaction and well-being at work that improve the overall utility of the OHS management.

Labor inspections are coordinated and directed by the Labor Inspection Boards that direct generally two kinds of inspections as occasional and planned. Occasional inspections occur in case of sudden needs. Planned inspections of workplaces should be scheduled at the beginning of the planning period based on the legal provisions. According to the schedule, inspectors are in a position to visit workplaces under their supervision without undue loss of time. “Visiting a workplace to monitor workplace hazards and to ensure compliance with regulation and legislation and writing a report concerning the visit” is defined as the audit task of inspectors. OHS inspectors' task assignment problem is complicated because of the technical and physical requirements of each task and inspectors' nonconflicting preferences with the both legal and ethical requirements of the problem.

In the World, there is comparatively extensive research on OHS legislation and regulation enforcement. Tompa et al. [17] and MacEachen et al. [18] presented a systematic review of qualitative research articles, which considers how OHS legislation and regulatory enforcement are planned and implemented. They mentioned that research studies on OHS legislation and enforcement have mainly drawn on quantitative methods and addressed the effectiveness and cost of various enforcement strategies. Geminiani et al.’s [19] and Niskanen's [20] studies are samples for the literature on OHS legislation and regulation enforcement, which was conducted with the aim of investigating the effectiveness and performance of the Department of Labor OHS Inspectorate. Limited studies about the role of OHS inspectors regarding the legislation related with psychosocial risk factors in workplaces mentioned in Johnstone et al.’s [21]. Wu [22] investigated the roles and functions of safety professionals. Chang et al. [23] developed a competency model of safety professionals. Recently, Wojtacka et al. [24] investigated the factors influencing veterinary food inspectors in Poland. Based on the survey consisting of 15 questionnaires conducted on 119 active veterinarians, the indicated problems are insufficient training, lack of preparation in coping with crisis situations danger while fulfilling professional duties because of different sources of hazards. Hagqvist et al. [25] interviewed 11 Swedish OHS inspectors and investigated their reflections on their bureaucratic role when supervising microenterprises. The results showed that OSH inspectors need organizational support to develop inspection models and enforcement styles tailored to microenterprises, as this could ease their work and contribute to better inspection outcomes. However, none of these studies is interested in OHS inspectors' task assignment.

OHS inspectors' task assignment is one of the significant levels of “personnel scheduling” problem that has been a focus area in manufacturing systems since its first introduced 1950s as labor/workforce scheduling. Today, effective personnel scheduling has great importance for organizations not only in manufacturing but also in the service industry. Activity/task assignment that consists in assigning to each period of each shift the activity or task to be performed by the employees is one of the significant levels of personnel scheduling [26]. Fairness, or equity, has also been incorporated in staff scheduling [[27], [28], [29]] to maintain the job satisfaction, which results in labor productivity. They attempt to distribute the workload fairly and evenly among personnel [30]. Equity is incorporated in mathematical models in either the objective function, for example, minimizing the variation in workload, or through the use of constraints, which provide lower and upper bounds on the workload [31].

A few studies have examined the relationship between work schedule and task quality from the behavioral science perspective. Dai et al. [32] found that healthcare workers become less compliant with handwashing rules over the course of their shift. Danziger et al. [33] examined the decision of eight judges and concluded that repeated decisions might have caused mental depletion. Ibanez and Toffel [34] examined how inspector schedules could introduce bias that erodes inspection quality by altering inspector stringency in food-safety inspections.

Ernst et al. [30] and Causmaecker et al.’s [35] classified personnel scheduling studies based on application areas. Van den Berghe et al. [36] discussed the classification methods in former review papers.

Nevertheless, none of these classifications indicated OHS inspectors' scheduling and/or OHS inspectorates. Only Ernst et al.’s [30] classification includes personnel scheduling studies in financial services, which is frequently called the *audit staff scheduling*. Although its technical requirements and the objective function(s)’ definition(s) are different from that of OHS inspectors' scheduling, managerial decisions regarding the both problems can be grouped into similar categories in terms of the length of the planning horizon and the planning periods, degree of aggregation of the audit tasks and degree of detail of the required information. Because the auditing firms are interested in the least cost schedule, the considered objectives in studies [[37], [38], [39]] constructed mainly on cost criterion. Mohamed [40] presents a state of art survey of the OR models developed for audit staff scheduling in financial services.

The aforementioned studies indicate two important results: one of which is that the existing research on OHS legislation and regulation enforcement mainly intensify on the effectiveness and cost of enforcement strategies, and performance evaluation of the Inspectorate Departments. The second result is that, the research on audit staff scheduling mainly restricted with financial services and accounting. Their aims make the objectives and the modeling requirement different from the OHS inspectorates' task assignment problem. In other words, OHS inspectors' task assignment problem is overlooked in the literature. The current study aims to solve the OHS inspectors' task assignment problem to maintain the application of OHS legislation and regulations efficiently by obtaining the most effective balanced task assignment among the labor inspectors.

## 2 Materials and methods

### 2.1 Preliminaries

OHS inspectors' task assignment problem is a multiple objective decision-making problem having the following objectives: to balance the total traveling distance of inspectors, to maximize the inspectors' preference score in total, and balancing the inspector's workloads in terms of the employee numbers in workplaces.

Work motivation and well-being of each inspector highly depends on the total traveling distance in a planning period and his/her satisfaction level related with his/her preferences on geographical location; moreover, these two criteria are prior to the workload defined in terms of the employee numbers in workplaces. Therefore, it is determined to be used a hierarchical solution approach, in which inspectors' preferences are considered. Inspiration comes from one of the multiobjective programming approach called The Method of Sequential Optimization or Lexicographic Method [41,42], in which decision maker ranks the objective functions according to some subjective priority so that a marginal improvement for any objective preempts arbitrarily large improvements in objectives of subsequent ranks. The Method of Sequential Optimization leads to nondominated solutions [43].

The mixed integer models of both stages (called Model 1 and Model 2) are also very close to the generalized assignment model formulation [44] when the number of inspections and workloads in terms of employee numbers are considered as resource capacities. The formulation of the generalized assignment problem is as follows:

(2.1) $$Minimize\;\underset{i\in I}{\sum }\underset{j\in J}{\sum }c_{ij}x_{ij}$$

Subject to

(2.2) $$\underset{i\in I}{\sum }b_{ij}x_{ij}\leq K_{j},\;j\in J,$$

(2.3) $$\underset{j\in J}{\sum }x_{ij}=1,\;i\in I,$$

(2.4) $$x_{ij}\in \left\{0,1\right\},\;i\in I,\;j\in J.$$

Assigning item i∈I to resource j∈J consumes b_ij_ units of resource j capacity and results in a cost of c_ij_. K_j_ is the available capacity of resource j. Decision variables x_ij_ are binary assignment variables. The objective function (2.1) minimizes the total assignment cost, (2.2) indicates the capacity restriction, and (2.3) guarantees that each item assigns to exactly to one resource. The special case in which b_ij_=1 ∀i,j; and the inequality in (2.2) is replaced with an equation sign is the appropriate formulation for the currently defined OHS inspectors' task assignment problem.

In the first stage, two objectives of Model 1 are balancing the total traveling distance of inspectors and maximizing the inspectors' preference scores in total respectively*.* Both objectives have equal weights. Balancing needs to minimize the total deviations from average traveling distance of committees. In the second stage, the objective of Model 2 is balancing the workloads in terms of the employee numbers in workplaces. Balancing objectives in two stages lead the analysts to use Goal Programming Approach [45,46]. Goal programming is one of the most powerful multiple objective decision making (MODM) approaches [47] because it has the functional representation of obtaining a nondominated solution [48]. This functional representation is directly related to the minimization of unwanted deviations from corresponding goals for each objective. In balancing objectives, decision maker wishes to alienate both positive and negative deviations, not the only one. In maximizing objectives, decision maker wishes to minimize the negative deviation between the achievements of the goal.

The details and versions of goal programming can be found in Refs. [[49], [50], [51], [52]]. The method has several variants. In the lexicographic goal programming approach, the decision maker (DM) must specify a lexicographic order for the goals in addition to the aspiration levels [53]. After the lexicographic ordering, the problem with the deviations as objective functions is solved lexicographically subject to the constraints. The algebraic formulation of lexicographic goal programming is as follows [51]:

Find **x=**(x_1_, x_2_, …,x_n_) so as to

(2.5) $$\text{Lexicographically}\;\text{minimize}\;a=\left({\text{g}}_{1}\left(d^{-},d^{+}\right),\&..,{\text{g}}_{\text{K}}\left(d^{-},d^{+}\right)\right)$$

Subject to

(2.6) $$f_{i}\left(x\right)+d_{i}^{-}-d_{i}^{+}=b_{i}\;\text{for}\;i=1,\&,Q,$$

(2.7) x∈X,

$$d^{-},d^{+}\geq 0$$

where **a** is the achievement function (2.5), g_k_(**d**^**−**^, **d**^**+**^) is the goal function at rank or priority level k and $b_{i}$ is the aspiration level associated with objective i. This model has K priority levels, and Q objectives. The achievement function **a** is an ordered vector of these K priority levels.$\;d_{i}^{-}$ and $d_{i}^{+}$are deviational variables, which represent the under and over achievement of the ith goal, respectively (2.6). **x** is the vector of decision variables to be determined. Any set of hard constraints (2.7) are placed, by convention, in the first priority level.

### 2.2 The steps of the proposed hierarchical solution approach

**Fig. 1:**
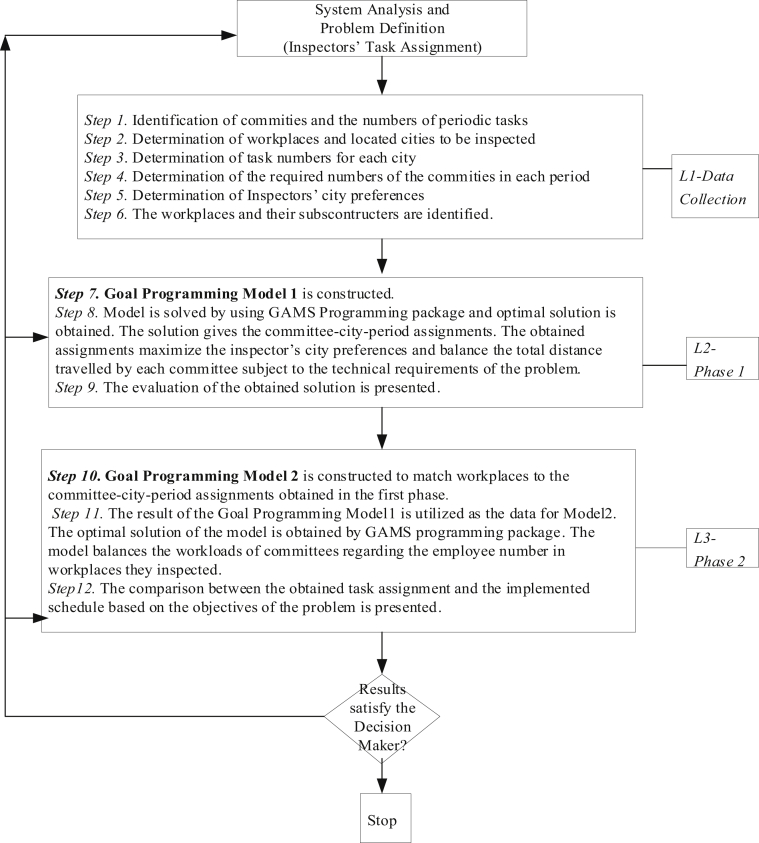
Flow chart of the steps of the proposed hierarchical solution approach. *L1. Data collection* constitutes the six steps. The data obtained by the first five steps are used in the Phase 1. The collected data in Step 6 is kept for Phase 2. *L2. The phase 1. Goal Programming Model 1* is constructed to obtain optimal committee-city-period assignments. *L3. The phase 2. Goal Programming Model 2* is constructed to match workplaces to the committee-city-period assignments obtained in the first phase. The last step (Step 12) is valid for the current study. In the future, it is performed as “the presentation of the obtain solution to the decision maker.”

The steps of the approach for solving the inspectors' task assignment problem are summarized in Fig. 1 as a flow chart in three levels, *L1, L2,* and *L3*.

## 3 Application

### 3.1 System analysis

Based on the regulations, the Labor Inspection Board of the Ministry of The Republic of Turkey executes the planning and implementation phases of inspections by the coordination and cooperation of five working-group-presidencies that are called with the big cities' names: Ankara, Adana, İzmir, Bursa, and İstanbul. Ankara is the largest group presidency in terms of both the number of provinces served and the total number of annual inspections. The Ankara Group serves 47 of 81 provinces. The other groups serve 15, 9, 6, and 4 provinces, respectively [54]. The number of OHS inspectors in each group presidency is different from each other. Currently, 1,005 inspectors were working at the Labor Inspection Board. Ankara Group Presidency carries out the audit task planning and assignment for whole inspectors. The board associates inspections with a certain number of projects that are mainly in the manufacturing or service industry. A committee usually consists of two OHS inspectors. Committees are assigned to a number of audit tasks in the planning period. The physical presence of the inspectors in the workplace is a legal requirement. Each committee monitors the assigned workplace based on the national legislation and prepares a report concerning the visit. Inspectors significantly contribute to the improvement in working conditions and the working environment. Hence, their employment procedure and in-service training are paid attention in the system. The employment procedure of inspectors starts with that the ministry conducts an examination to select the sufficient applicants as assistant inspectors. After a 30-year job-training period in that position, only the passers of the proficiency examination qualify to be a full inspector. After a 10-year work on the field, an inspector becomes a head inspector. Inspectors participate in continuing learning programs and national and international conferences on occupational safety and health during the work. They legally cannot work in any other profession and work on a full-time basis. Inspectors' nonconflicting preferences with the both legal and ethical requirements of the problem are taken into consideration on the contrary to the traditional approach.

### 3.2 Problem statement

This study concentrates on one of the planned OHS inspections carried out by the board in the Production of Mineral Products with the project code P2. Mineral products comprise of the production of cement, ceramics, glass, and lime. Mineral processing is also known as nonmetallic mineral product manufacturing, which is characterized by the transformation of naturally occurring minerals such as sand, gravel, stone, clay, limestone, and silica in the form of dust to the desired form through an energy-intensive process. The products range from bricks and tiles to glass and tableware. Utilized processes include heating, melting, grinding, mixing, cutting, shaping, and honing. The requirements of the considered project P2 are widespread and cover others requirements. Hence, it was chosen as a representative for the other projects. In which 20 inspectors are planned to audit 328 workplaces for 9 months. To maintain the balanced task assignment, the analyst faced three challenges. The first one regards to the workloads: the potential workload capacity of a committee is defined as the number of tasks they are allowed to perform in a period and a task is a workplace visit. However, the workload of an audit task shows differences from a workplace to another because of their sizes. The number of employees in each workplace is used as an indicator of its size. The second challenge regards the workplaces' distances from the provincial centers. Some workplaces are closer to the centers, whereas others are located in the districts. The third challenge, is that the workplaces and their subcontractor(s) appear(s) to be separate enterprise(s)/business(es) according to the lists declared by the Social Security Institution [55]. Such workplaces should be identified and be assigned to the same inspection committee in the same period and besides, visiting a subcontractor should still be defined as a task.

The inspection tasks of workplaces are needed to assign to committees in a balanced manner with the following nine assumptions listed from A1 to A9 as follows: A1. The inspections are carried out from the beginning of February to the end of November. A2. Nine-month time zone is divided into five periods. Each period (j = 1, 2, …,5) composed of 2 months (February–March, April–May, June–July, September–October) except the last one. The last period constitutes only the month November. A3. In the first two periods (j = 1, 2), only eight committees are available to assign to tasks and for the last three periods (j = 3, 4, 5), whole 10 committees are available to assign to tasks. A4. Each committee has to work in each period. If the total number of workplaces in a city is not sufficient to constitute a task in each period, then the workplaces in that city should be inspected in distinct periods. A5. Each city should be visited in each period at least one time. A6. A committee is allowed to assign to same city at most two times within five periods. A7. The total distance that is traveled by each committee should be balanced. A8. The inspectors' preferences related with cities will be taken into account. A9. The same committee inspects a workplace and its subcontractor in the same period.

### 3.3 L1—data collection

Step 1 In the project, 20 inspectors (10 committees) audit 328 workplaces for 9 months. The total number of workplaces is determined by considering the number of available inspectors employed in the Ministry and their potential workload capacity. For each committee, the number of tasks is determined as "8" in Periods 1, 2, 3, and 4; and it is determined as "4" in the Period 5.
Step 2 An inspection of a workplace is defined as a task. The total number of tasks within a 1-year planning period, the number of available committees and their available workloads are presented in the Table 1.

**Table 1:** Total number of tasks within a 1-year planning period

Period number (*j*)	Month(s)	The number of available committees	Allowed task number per committee (workload of each committee in each period)	Total number of workload capacity in each period
1	February–March	8	8	8 × 8 = 64
2	April–May	8	8	64
3	June–July	10	8	80
4	September–October	10	8	80
5	November	10	4	40
Total number of tasks within the 1-year planning period				328
Step 3 The number of the workplaces to be inspected in city, and their total employment numbers and the average number of employments per workplace are presented in Table 2.

**Table 2:** The number of workplaces and their employment numbers in each city

*k*	City (*k*)	The number of workplaces to be inspected in each city, *T*_*k*_	The total number of employees in workplaces	The average number of employees per workplace
1	Denizli	48	1,652	34
2	Uşak	12	1,632	136
3	Muğla	44	498	11
4	Aydın	48	1,523	32
5	Kütahya	104	7,235	70
6	Kırklareli	24	2,973	124
7	Bilecik	48	8,141	170
Total		328	23,654	72
Step 4 The number of inspections to be conducted in each city based on the task numbers of each committee in each period is presented in Table 3. “*T*_*k*_” presents the total number of workplaces in city k and each workplace is corresponds to a task/an inspection. “*A*_*k*_”, is defined as the number of inspections to be conducted in the first four periods in city k. “*S*_*k*_” is defined as the number of inspections to be conducted in the last period in city k. Table 3 also presents the distance between each city and the corresponding main center in the last column based on the General Directorate of Highways' Internet page [56].

**Table 3:** The number of inspections to be conducted in each city

*K*	City	The number of workplaces (*T*_*k*_)	8*A*_*k*_ +4*S*_*k*_ = *T*_*k*_		*D*_*k*_ (km) distance of city k to the main center
			A_k_	S_k_	
1	Denizli	48	5	2	475
2	Uşak	12	1	1	368
3	Muğla	44	5	1	620
4	Aydın	48	5	2	598
5	Kütahya	104	12	2	311
6	Kırklareli	24	3	0	664
7	Bilecik	48	5	2	315
Total		328	36	10	3,351 {Average distance = (3,351/7) = 478.71}
Step 5 Inspectors' city preferences are investigated by interviews and their common decision is used as each committee's preference score. Each committee is required to make preferential orderings for each city for each period. Because there are seven cities to be audited, the scoring from the most preferred to least, takes 7 to 1 scores. The committees' ordered city preferences in each period are given in Table 4.

**Table 4:** Committees' ordered city preferences in each period

Committee (i)	Period (j)	Preference order/score City						
		1/7	2/6	3/5	4/4	5/3	6/2	7/1
1	1	Aydın	Muğla	Denizli	Kırklareli	Kütahya	Uşak	Bilecik
	2	Denizli	Kırklareli	Aydın	Muğla	Uşak	Kütahya	Bilecik
	3	Kırklareli	Denizli	Aydın	Bilecik	Uşak	Kütahya	Muğla
	4	Bilecik	Uşak	Denizli	Kırklareli	Aydın	Kütahya	Muğla
	5	Uşak	Aydın	Bilecik	Kırklareli	Kütahya	Denizli	Muğla
2	1	Muğla	Uşak	Denizli	Bilecik	Kırklareli	Kütahya	Aydın
	2	Kütahya	Bilecik	Kırklareli	Denizli	Uşak	Aydın	Muğla
	3	Kütahya	Uşak	Denizli	Muğla	Aydın	Kırklareli	Bilecik
	4	Uşak	Denizli	Kütahya	Muğla	Kırklareli	Bilecik	Aydın
	5	Kütahya	Kırklareli	Uşak	Denizli	Aydın	Muğla	Bilecik
3	1	Uşak	Muğla	Denizli	Aydın	Kırklareli	Kütahya	Bilecik
	2	Bilecik	Uşak	Denizli	Aydın	Kırklareli	Kütahya	Muğla
	3	Uşak	Denizli	Kütahya	Kırklareli	Muğla	Aydın	Bilecik
	4	Muğla	Uşak	Denizli	Kütahya	Bilecik	Aydın	Kırklareli
	5	Kütahya	Denizli	Kırklareli	Bilecik	Muğla	Uşak	Aydın
4	1	Denizli	Kütahya	Kırklareli	Bilecik	Uşak	Muğla	Aydın
	2	Aydın	Uşak	Kırklareli	Muğla	Kütahya	Bilecik	Denizli
	3	Kırklareli	Bilecik	Denizli	Uşak	Kütahya	Aydın	Muğla
	4	Muğla	Uşak	Bilecik	Aydın	Kütahya	Denizli	Kırklareli
	5	Uşak	Kütahya	Aydın	Denizli	Kırklareli	Muğla	Bilecik
5	1	Aydın	Denizli	Bilecik	Muğla	Kütahya	Uşak	Kırklareli
	2	Kütahya	Aydın	Muğla	Kırklareli	Bilecik	Uşak	Denizli
	3	Kırklareli	Uşak	Aydın	Denizli	Muğla	Kütahya	Bilecik
	4	Denizli	Bilecik	Aydın	Muğla	Uşak	Kütahya	Kırklareli
	5	Muğla	Aydın	Kütahya	Uşak	Denizli	Kırklareli	Bilecik
6	1	Kütahya	Uşak	Muğla	Kırklareli	Aydın	Bilecik	Denizli
	2	Kırklareli	Kütahya	Muğla	Denizli	Uşak	Bilecik	Aydın
	3	Muğla	Uşak	Kırklareli	Aydın	Kütahya	Bilecik	Denizli
	4	Kütahya	Aydın	Muğla	Kırklareli	Bilecik	Uşak	Denizli
	5	Uşak	Muğla	Kırklareli	Kütahya	Denizli	Bilecik	Aydın
7	1	Aydın	Uşak	Bilecik	Muğla	Kütahya	Denizli	Kırklareli
	2	Kütahya	Kırklareli	Bilecik	Denizli	Aydın	Uşak	Muğla
	3	Kütahya	Aydın	Kırklareli	Denizli	Uşak	Bilecik	Muğla
	4	Muğla	Kırklareli	Bilecik	Denizli	Aydın	Uşak	Kütahya
	5	Uşak	Denizli	Bilecik	Kütahya	Kırklareli	Muğla	Aydın
8	1	Denizli	Kütahya	Kırklareli	Muğla	Aydın	Bilecik	Uşak
	2	Bilecik	Muğla	Kırklareli	Denizli	Aydın	Uşak	Kütahya
	3	Kütahya	Kırklareli	Bilecik	Denizli	Aydın	Uşak	Muğla
	4	Kırklareli	Uşak	Bilecik	Denizli	Kütahya	Muğla	Aydın
	5	Muğla	Kırklareli	Kütahya	Denizli	Uşak	Aydın	Bilecik
9	3	Denizli	Uşak	Bilecik	Kütahya	Kırklareli	Aydın	Muğla
	4	Aydın	Bilecik	Kırklareli	Denizli	Muğla	Uşak	Kütahya
	5	Kırklareli	Muğla	Bilecik	Denizli	Aydın	Kütahya	Uşak
10	3	Aydın	Kırklareli	Kütahya	Denizli	Muğla	Uşak	Bilecik
	4	Bilecik	Uşak	Aydın	Muğla	Kütahya	Denizli	Kırklareli
	5	Kütahya	Kırklareli	Bilecik	Denizli	Aydın	Uşak	Muğla

The target value for the traveling distance for the first five periods is denoted by TDp1-5 and it is calculated as 2,394 km by multiplying the average distance that a committee traveled in one period (478.71 km calculated in Table 3) by five periods. The target value for the traveling distance for the last three periods is denoted by TDp3-5 and it is calculated in a similar manner as 1,436 km. For the distance calculations, only one-way traveling directions are considered.

Step 6 Goal Programming Model 2 aims to match workplaces/tasks to the committee-period-city assignments obtained in the first phase. Before constructing the Model 2, the workplaces that have to be inspected by the same committee in the same period based on the assumption 9 are identified and presented in Table 5 in one cluster.

**Table 5:** The workplaces that have to be inspected by the same committee in the same period based on the assumption 9

Cluster $c_{t_{k}}$	Workplaces' order numbers (*t*_*k*_) for each city						
	*k* = 1 Denizli	*k* = 2 Uşak	*k* = 3 Muğla	*k* = 4 Aydın	*k* = 5 Kütahya	*k* = 6 Kırklareli	*k* = 7 Bilecik
1	1,7,10,11,12	1,3	1,31	15,20,37,43	1,4	2,3	1,7,29
2	13,15,46	2,8	2,18,30	41,47	2,6,52	5,11,17,18,19,23	3,17,30,36
3	17,41	5,11	4,22	44,45	3,5,8,11,15,22,85	10,22	5,13,14,15,22,24,26,37
4	22,48	6,7	5,13		7,16	22,24	11,23,31,32,33,38,39,400
5	28,38		8,15		9,28,65		41,42,43,44,46,47
6			14,19		10,20,22,23,26,30		45,48
7			22,34		14,17,59		
8					18,62		
9					32,84		
10					36,40		
11					39,44		
12					45,50		
13					57,71		
14					70,104

These workplaces are not only constitute by the subconstructers, but also constitute by the different workplaces having the same address and the same workplaces having different addresses in the same city. In city Denizli (*k* = 1), there are 34 workplaces (*T*_*1*_ = 34) to be inspected (Table 2). The workplaces in each city denoted by “*t*_*k*_”. In Table 5, for *k* = 1 there are five clusters. Each cluster constitutes different numbers of workplaces that have to be inspected by the same committee in the same period. The first cluster ($c_{t_{1}}$) constitutes workplaces 1, 7, 10, 11, and 12; $\;c_{t_{1}}$ = {1, 7, 10, 11, 12}. The inspection of these five workplaces corresponds to five tasks.

### 3.4 L2—Phase 1. Goal Programming Model 1

*Index sets:*

*i* index for committees, for all *i* = 1,2, …, 10
*j* index for periods, for all *j* = 1, 2, 3, 4, 5
*k* index for cities, for all *k* = 1, 2, 3, 4, 5, 6, 7.

*Decision variables:*

*X*_*ijk*_ = If committee i visit city k in period j, then 1; otherwise 0
$d_{i}^{-}=\text{Negative deviation in constraint }i,\;\forall i$
$d_{i}^{+}=\text{Positive devaition in constraint }i,$∀*i*
$d_{ik}^{-}=\text{Negative deviation in constraint }ik\;,\;\forall ik$
$d_{ik}^{+}=\text{Positive devaition in constraint }ik,$ ∀*ik*.

*Parameters:*

*P*_*ijk*_ = The score number attained by the committee *i*, in period *j*, for city *k* (Table 3).
*D*_*k*_ = The traveling distance between city *k* and the main center Ankara (Table 4).
*TD*_*p1-5*_ = 2,394 km (The target traveling distance for each committee, where ∀*i*∖{9,10} for five periods, its calculation is explained in Section 3, Step 5).
*TD*_*p3-5*_ = 1,436 km (The target traveling distance for each committee where *i* = {9, 10} for three periods, its calculation is explained in Section 3, Step 5).
*A*_*k*_ = The number of inspections to be conducted in the first four periods in city *k* (Table 4).
*S*_*k*_ = The number of inspections to be conducted in the last period in city *k* (Table 4).
*TS* = 322 point (The total ideal score, which is calculated as the multiplication of the maximum total score and the total task number, 7 × 46 = 322).
*TTD* = 33,510 km (The total ideal traveling distance, which is calculated by the multiplication of average traveling distance and the total committee number, 3351 × 10 = 33,510 km).

Objective Function Phase 1

(3.1) $$Min\;z=\;\underset{i}{\sum }\left(\left(d_{i}^{-}+d_{i}^{+}\right)/TTD\right)+\underset{j,k}{\sum }\left(d_{ijk}^{-}/TS\right)$$

Subject to

(3.2) $$\underset{i}{\sum }\overset{4}{\underset{j=1}{\sum }}X_{ijk}=A_{k},\;\forall k$$

(3.3) $$\underset{i}{\sum }X_{i5k}=S_{k},\;\forall k/\left\{6\right\}$$

(3.4) $$\underset{k}{\sum }X_{ijk}=1,\;\forall i;\forall j$$

(3.5) $$\overset{8}{\underset{i=1}{\sum }}\underset{k}{\sum }X_{ijk}\geq 1,\;\forall j\in \left\{1,2\right\}$$

(3.6) $$\overset{10}{\underset{i=1}{\sum }}\underset{k\setminus \left\{6\right\}}{\sum }X_{ijk}\geq 1,\;\forall j\in \left\{3,4,5\right\}$$

(3.7) $$\sum_{j}\sum_{k}D_{k}X_{ijk}+d_{i}^{-}-d_{i}^{+}=TD_{p1-5},\forall i\setminus \left\{9,10\right\}$$

(3.8) $$\overset{5}{\underset{j=3}{\sum }}\overset{7}{\underset{k=1}{\sum }}D_{k}X_{ijk}+d_{i}^{-}-d_{i}^{+}=TD_{p3-5},\;\forall i\in \left\{9,10\right\}$$

(3.9) $$\overset{10}{\underset{i=9}{\sum }}\overset{2}{\underset{j=1}{\sum }}X_{ijk}=0,\;\forall k$$

(3.10) $$\overset{2}{\underset{j=1}{\sum }}P_{ijk}*X_{ijk}+d_{ijk}^{-}=7,\;\forall i/\left\{9,10\right\};\;\forall k$$

(3.11) $$\overset{5}{\underset{j=3}{\sum }}P_{ijk}*X_{ijk}+d_{ijk}^{-}=7,\;\forall i;\;\forall k$$

(3.12) $$\underset{i}{\sum }\underset{j}{\sum }X_{ijk}\leq 2,\;\forall k$$

(3.13) $$X_{ijk\;}\varepsilon \left\{0,\;1\right\},\;\forall i,j,k$$

(3.14) $$d_{i}^{-},\;d_{i}^{+},d_{ijk}^{-},\;d_{ijk}^{+}\geq 0,\;\forall i,j,k$$

The objective function (3.1) of the problem is to minimize the total of positive and negative deviations from average traveling distance of committees and the total of negative deviations from the total preferencescore attained to cities. For unit recovery on distance (km) and score (17 scale), the first term in the objective function is divided by the total ideal distance and the second term in the objective function is divided by the total ideal score. Equations (3.2), (3.3) restrict the number of inspections to be conducted in periods *j* = 1 to 4, and the last period (*j* = 5), respectively, in city *k*. Equation (3.4) maintains that each committee is charged in only one city in each period. Equations (3.5), (3.6) provide that each city be visited at least once in each period. These constraints are constructed for the first two periods and the last three periods, respectively. Total employee number in the workplaces in the city numbered 6 are not enough to constitute a task for each period. Equation (3.6) maintains that workplaces in City 6 are not inspected in the last period, because S_k_ is calculated as “0” for *k* = 6 in Table 3. Equations (3.7), (3.8) are goal constraints related with the traveling distance of each committee in each period. Equation (3.7) restricts that the total traveling distance of each committee, where *i*∈{1,2,..,8}; is equal to the target traveling distance TD_p1-5_ in periods 1 and 2. Equation (3.8) restricts that the total traveling distance of committees 9 and 10 in periods *j* = 3, 4, 5 is equal to the target traveling distance D_p3-5_. Equation (3.9) provides that the committees numbered *i* = 9 and 10 are not in charge in periods *j* = 1 and 2. Equations (3.10), (3.11) are goal constraints related with the preference score of each committee. Equation (3.10) is written for the first eight committees, which are charged in periods *j* = 1 and 2. Because each committee is charged in only one city in only one period (Equation (3.4)), Equation (3.10) restricts the total preference score with the maximum score value as seven points. Similarly, Equation (3.11) is written for whole 10 committees, which are charged in periods *j* = 3,4, and 5 and it is restricts the total preference score with the maximum score value as seven points. Equation (3.12) allows that each committee visits a city at most two times within five periods. Equation (3.13) presents 0-1 integer constraints. Equation (3.14) presents the positivity restrictions for variables.

The obtained committee-period-city assignments based on the optimal solution of Phase 1 are summarized in Table 6, which indicates that the majority of preference scores are "7" based on the solution, which means that the majority of committees are assigned cities where they wish to visit. Minimization of the positive and negative deviations from the target distance leads the model to reach balanced traveling distance for each committee. Furthermore, the minimization of the negative deviations from the preference score leads the model to achieve the city assignments having the maximum score in total.

**Table 6:** Phase 1 Solution Summary, committee-period-city assignments based on the values of decision variable X_ijk_ and the preference score (P_ijk_) of each city

Committee (*i*)	Period (*j*) (*k*, (city Name))/Preference score (*P*_*ijk*_)					Total score for each committee
	1 (February–March)	2 (April–May)	3 (June–July)	4 (September–October)	5 (November)	
1	4 (Aydın)/7	1 (Denizli)/7	1 (Denizli)/6	7 (Bilecik)/7	4 (Aydın)/6	33
2	3 (Muğla)/7	5 (Kütahya)/7	5 (Kütahya)/7	2 (Uşak)/7	1 (Denizli)/4	32
3	1 (Denizli)/5	7 (Bilecik)/7	5 (Kütahya)/5	5 (Kütahya)/4	1 (Denizli)/6	27
4	5 (Kütahya)/6	4 (Aydın)/7	7 (Bilecik)/6	3 (Muğla)/7	5 (Kütahya)/6	32
5	7 (Bilecik)/5	5 (Kütahya)/7	6 (Kırklareli)/7	1 (Denizli)/7	4 (Aydın)/6	32
6	5 (Kütahya)/7	6 (Kırklareli)/7	3 (Muğla)/7	5 (Kütahya)/7	2 (Uşak)/7	35
7	4 (Aydın)/7	5 (Kütahya)/7	5 (Kütahya)/7	3 (Muğla)/7	7 (Bilecik/)/5	33
8	5 (Kütahya)/6	3 (Muğla)/6	5 (Kütahya)/7	6 (Kırklareli)/7	3 (Muğla)/7	33
9			1 (Denizli)/7	4 (Aydın)/7	7 (Bilecik)/5	19
10			4 (Aydın)/7	7 (Bilecik)/7	5 (Kütahya)/7	21
Total score						297

### 3.5 L3—Phase 2. Goal Programming Model 2

Considering the differences between the number of workplaces and employee numbers in a city, the analyst defines an objective function to balance the workloads based on the size of the workplaces in a city. The objective function is to minimize the total deviations from the average employee numbers in workplaces to be inspected in each city. Notations used in this model are as follows:

*Index sets:*

*i* index for committees, for all *i* = 1,2, …, 10
*j* index for periods, for all *j* = 1, 2, 3, 4, 5
*k* index for cities, for all *k* = 1, 2, 3, 4, 5, 6, 7
*t*_*k*_ index for workplace t in city k for all *t*_*k*_ = 1,2, …, *T*_*k*_
$c_{t_{k}}$ index for workplace clusters in city k for all $c_{t_{k}}$ given in Table 5.

*Decision variables:*

$X_{\left(ijk\right)t_{k}}$ = If “committee *i* assigned to city *k* in period *j* in phase 1”, is matched to the workplace *t* in the same city *k*, then 1; otherwise 0
$d_{\left(ijk\right)}^{-}=\text{Negative deviation in constraint }\left(ijk\right)$
$d_{\left(ijk\right)}^{+}=\text{Positive devaition in constraint }\left(ijk\right)$

*Parameters:*

X = [*X*_*ijk*_ ]; 0–1 assignment matrix, which is obtained at the end of Phase 1; *X*_*ijk*_ = 1, if committee *i* assigned to city *k* in period *j*; 0 otherwise.
*NE*_*tk*_: The number of employees in workplace “*t*” in city “*k*”.
*AvrNE*_*k*_: The average number of employees in whole workplaces in city “*k*”.
*WL*_*j*_: The number of workloads of each committee in period j, in Periods 1 to 4, it is equal to 8; in Period 5, it is equal to 4.
*T*_*k*_: The number of workplaces to be inspected in city *k* (i.e., the number of tasks in city *k*)

$$\left|c_{t_{k}}\right|:\text{Cardinality}\;\text{of}\;c_{t_{k}}\;\text{cluster}\;\forall t,k$$

Objective Function Phase 2

(3.15) $$Min\;z=\;\underset{i,j,k}{\sum }\left(d_{\left(ijk\right)}^{-}+d_{\left(ijk\right)}^{+}\right)$$

Subject to

(3.16) $$\underset{i}{\sum }\underset{j}{\sum }X_{\left(ijk\right)t_{k}}=1,\;\forall k,\;t_{k}$$

(3.17) $$\underset{k}{\sum }\underset{t_{k}}{\sum }X_{\left(ijk\right)t_{k}}=WL_{j},\;\forall i,j$$

(3.18) $$\underset{t_{k}\in c_{t_{k}}}{\sum }X_{\left(ijk\right)t_{k}}=\left|c_{t_{k}}\right|,\;\forall i,j,k$$

(3.19) $$\underset{t_{k}}{\sum }X_{\left(ijk\right)t_{k}}NE_{t_{k}}+d_{\left(ijk\right)}^{-}-d_{\left(ijk\right)}^{+}=WL_{j}AvrNE_{k},\;\forall i,j,k$$

(3.20) $$X_{\left(ijk\right)t_{k}}\varepsilon \;\left\{0,1\right\}\;\forall i,j,k,t$$

(3.21) $$d_{\left(ijk\right)}^{-},\;d_{\left(ijk\right)}^{+}\geq 0,\;\forall \left(ijk\right)$$

*X*_*ijk*_ assignments are at hand and they are obtained from the first-phase solution. The objective function (3.15) is to minimize the total deviations of the total number of employees from the average number of employees in workplaces inspected by each committee in each city in the related period. Equations (3.16) restricts that each workplace in a city is visited only one committee. Equation (3.17) restricts the number of tasks in each city in each period with the number of workloads of each committee in related periods. Equation (3.18) restricts that workplaces and their subcontractor(s) are assigned to same committee in each city in each period. Equation (3.19) restricts that the total number of employees in workplaces inspected by each committee in each city with its target value in the related period. Equation (3.20) presents 0–1 integer constraints. Equation (3.21) presents the positivity restrictions for variables.

The optimal objective function value is obtained as 10,872 employees. Minimization of the positive and negative deviations of the total number of employees from its target value leads the model to reach a workload balance beyond the workload balance provided by assigning the same number of audit task to each committee. Committee-city-period-workplace assignments are determined based on *X*_*(ijk)tk*_ variables, which take value “1” in the optimal solution and they are represented as a clear schedule for the ministry and the inspectors in Table 7.

**Table 7:** Phase 2 Solution Summary, Committee-city-period-workplace assignments

Committee (i)	Period (j)									
	1 (February–March)		2 (April–May)		3 (June–July)		4 (September–October)		5 (November)	
	City (k)	Workplace (t_k_)	City	Workplace	City	Workplace	City	Workplace	City	Workplace
1	4 (AYDIN)	7, 8,10, 12, 13, 14, 23, 27	1 (DENİZLİ)	6, 19,22, 33,34, 37, 42, 43	1 (DENİZLİ)	1, 7,10, 11,12, 44, 45, 47	7 (BİLECİK)	1, 7,20, 22,27, 29, 34, 35	4 (AYDIN)	5, 16, 18, 26
2	3 (MUĞLA)	8,9,12, 15,17,23, 29,35	5 (KÜTAHYA)	7,16,19, 36,40,66, 78,79	5 (KÜTAHYA)	3,5,8, 11,15,22, 85,88	2 (UŞAK)	1,3,4 6,7,9, 10,12	1 (DENİZLİ)	8,14, 32,36
3	1 (DENİZLİ)	2,17,27, 29,30,39, 40,41	7 (BİLECİK)	2,4,41, 42,43,44, 46,47	5 (KÜTAHYA)	1,4,93, 99,100,101, 102,103	5 (KÜTAHYA)	18,37,38, 58,62,74, 75,76	1 (DENİZLİ)	5,23, 24,26
4	5 (KÜTAHYA)	14,17,32, 35,45,50, 59,84	4 (AYDIN)	4,9,17, 19,22,22, 24,33	7 (BİLECİK)	11,23,31, 32,33,38, 39,40	3 (MUĞLA)	2,10,18, 30,32,33, 40,43	5 (KÜTAHYA)	29,42, 68,72
5	7 (BİLECİK)	3,8,17, 19,25,28, 30,36	5 (KÜTAHYA)	2,6,52, 94,95,96, 97,98	6 (KIRKLARELİ)	1,5,11, 13,17,18, 19,23	1 (DENİZLİ)	3,9,18, 22,25,31, 35,48	4 (AYDIN)	6,11, 29,35
6	5 (KÜTAHYA)	9,28,39, 44,61,63, 64,65	6 (KIRKLARELİ)	4,6,7, 8,9,10, 12,22	3 (MUĞLA)	6,11,14, 16,19,24, 25,26	5 (KÜTAHYA)	27,47,70, 73,86,90, 91,104	2 (UŞAK)	2,5, 8,11
7	4 (AYDIN)	3,15,20, 28,30,31, 37,43	5 (KÜTAHYA)	43,46,67, 69,77,81, 82,83	5 (KÜTAHYA)	10,20,22, 23,26,30, 53,54	3 (MUĞLA)	1,4,22, 1,39,41, 42,44	7 (BİLECİK)	10,12, 16,18
8	5 (KÜTAHYA)	13,25,31 33,34,49, 55,56	3 (MUĞLA)	5,7,13, 20,22,27, 28,34	5 (KÜTAHYA)	12,24,57, 71,80,87, 89,92	6 (KIRKLARELİ)	2,3,14, 15,16,20, 22,24	3 (MUĞLA)	3,36, 37,48
9					1 (DENİZLİ)	4,13,15, 16,20,28, 38,46	4 (AYDIN)	2,25,32, 34,36,38, 39,40	7 (BİLECİK)	6,9, 45,48
10					4 (AYDIN)	1,41,42, 44,45,46, 47,48	7 (BİLECİK)	5,13,14, 15,22,24, 26,37	5 (KÜTAHYA)	41,48, 51,60

## 4 Discussion

In the beginning of the working period, the inspector task assignment was constructed manually without performing a scientific approach and was implemented during the year. The comparisons of the implemented schedule and the schedule obtained by the proposed approach are made based on the preference score, the total traveling distance of each committee and the total number of employees in workplaces inspected by each committee, which are the objective criteria of the considered problem.

Based on the implemented schedule, committees' preference scores are calculated in Table 8 using the score values coming from the data-collection-phase of the proposed method and presented in Table 4.

**Table 8:** The committees' preference scores in the implemented schedule

Committee (i)/Period(j)	City/preference score					
	1 (February–March)	2 (April–May)	3 (June–July)	4 (September–October)	5 (November)	Total Score
1	Kütahya/3	Aydın/5	Kütahya/2	Denizli/5	Bilecik/5	20
2	Aydın/5	Kütahya/7	Denizli/5	Kütahya/5	Bilecik/1	23
3	Kütahya/2	Bilecik/7	Aydın/2	Denizli/5	Kütahya/7	23
4	Bilecik/4	Kütahya/3	Muğla/1	Aydın/4	Kütahya/6	18
5	Kütahya/3	Denizli/1	Aydın/5	Bilecik/6	Muğla/7	22
6	Denizli/1	Kütahya/6	Bilecik/2	Muğla/5	Aydın/1	15
7	Muğla/4	Uşak/2	Kütahya/7	Kırklareli/6	Aydın/1	20
8	Kırklareli/5	Muğla/6	Bilecik/5	Kütahya/3	Denizli/4	23
9			Kütahya/4	Muğla/3	Uşak/1	8
10			Kırklareli/6	Kütahya/3	Denizli/4	13
	27	37	39	45	37	185

The total preference score in the implemented schedule is calculated as 185 points, which is rather less than the total score (297 points) in the obtained optimal schedule. If it is assumed that whole committees could visit their firstly preferred cities, then the total ideal score (*TS*) was 332 points. Reaching this score is impossible because of the other restrictions in the mathematical model. However, the obtained schedule comes 89.46% close to this utopic score. Hence, the assignment based on the obtained schedule is satisfactory in terms of the preferences of committees.

The total distances traveled are equal and 20,518 km in both implemented and the obtained schedules. The total distances traveled by each committee obtained in Phase 1 and obtained in the implemented schedule are presented in Table 9. Because the claim of the proposed approach is to obtain a balanced schedule based on the total distance traveled by each committee; standard deviation, σ, is used as an indicator to check the balance in the traveled distance between each committee. The target traveling distances are different from each other for committees i = 1,2, …,8 and for i = 9,10. Therefore, the standard deviation, $\sigma_{i=1,2,\ldots ,8},$ of the distances traveled by committees 1-–8 in the obtained schedule is 46 and it is smaller than that of in the implemented schedule that is 265. The small standard variation indicates the balance between committees in their total distances traveled by. The standard deviation, $\sigma_{i=9,10},$ of the distances traveled by committees 9–10 in both schedules are close to each other.

**Table 9:** The comparisons based on the total traveling distance (km) and based on the total number of employees in workplaces

Committee (i)	The implemented schedule	The obtained schedule phase 1 solution summary	The implemented schedule	The obtained schedule phase 2 solution summary
	The distance traveled by committee i	The total distance traveled by committee i	The number of employees	The number of employees
1	2,010	2,461	3,748	3,187
2	2,010	2,085	2,656	3,275
3	2,010	1,887	1,205	4,058
4	2,155	2,255	4,500	1,126
5	2,319	2,363	2,209	4,468
6	2,319	2,274	1,002	1,256
7	2,561	2,255	3,017	1,500
8	2,385	2,526	1,690	2,065
9	1,299	1,388	584	1,227
10	1,450	1,224	3,043	1,502
δ for i = 1 to 8	265,2	46,0		
δ for i = 9 to 10	106,8	116,0		

The total number of employees is equal and 23,654 in both implemented and the obtained schedules. The claim of the proposed approach is to obtain a balanced schedule based on the workloads. The numbers of employees supervised in the inspected workplaces for each committee determined in Phase 2 and obtained in the implemented schedule are in last two columns on the right in Table 9. The standard deviation is not used for the employee number criterion. Because, the mathematical model in Phase 2, which minimizes the total deviations, defines each deviation based on the *considered city's* average employee number. To figure out the unbalancing in workloads in the implemented schedule, it is remarkable to note that the numbers of employees which is supervised by committees 9 and 10, which are 584 and 3,043, respectively, in Table 9 column 4.

The comparisons of the implemented schedule and the schedule obtained by the proposed approach show that the proposed one gives a significantly more effective task assignment based on the preference score, the total traveling distance of each committee and the total number of employees in workplaces inspected by each committee, which are the objective criteria of the current problem.

Although there is no study on OHS inspectors' task assignment problem in the literature, we can compare the current study with previous studies based on two factors. First one is fairness, justice or equity that is incorporated in mathematical models in either the objective function, for example, minimizing the variation in workload, or through the use of constraints, which provide lower and upper bounds on the workload [31]. From this point of view, the current study follows the first approach. The workload balance is maintained by the minimization of the variations from total traveling distance and total number of employees in workplaces defined mathematically in the objective functions.

The second factor is human preferences that are added to a mathematical model “before,” “during,” or “after” optimization process in multiobjective decision making as preferences of decision makers. These methods are called as priori, interactive, and posteriori methods [53]. In multiple attribute utility theory [41], the preferences are scored to express mathematically. The study used the multiple attribute utility theory to express inspectors' preferences and insert them into the mathematical model to maximize the total score.

It is worth mentioning two studies [57,58] indicating similar concerns of and compare them with the current study. Yi and Wang [57] investigated the task assignment of laborers in a project accounting by considering equity in terms of job completion time and the total extra energy expenditure. Although the current study able to find optimal solution for each phase, Yi and Wang [57] proposed a heuristic solution algorithm and find an approximate solution and did not consider the labors' task preferences in their model.

Nahand et al. [58] developed a multiple objective nurse scheduling model to minimize the human error in health care system. Objectives in the model are to maximize nurses' preference score and to minimize penalty cost of assigning a nurse to late-night shifts and to weekend shifts. Optimal solution is obtained by the weighted-sum method. Unlike the current study, equity was incorporated in the model through the use of constraints, which provide lower and upper bounds on the workload.

## 5 Conclusion

This study considers OHS inspectors' task assignment problem and proposes a solution approach. Human factor in the problem necessitates considering not only the technical and physical requirements but also the psychosocial factors that have impact on inspector accuracy.

Inspector accuracy is associated with basic individual abilities (nontechnical human issues); organizational factors (instructions, training, physical conditions); and interpersonal relations and social relations [59]. Without denying the importance of these factors individually, the actual limits in a working situation are set by the psychosocial laterality of these factors. Psychosocial risks are now widely acknowledged as a priority in OHS [60]. Mental and physical health problems associated with workplace originated from psychosocial risk factors are a significant, well-documented health issue [61]. Labor Inspectors are government representatives and also employees indeed and to maintain their well-being at work and job satisfaction should be better considered as significant psychosocial risk factors in their workplace.

Job satisfaction is directly affected by the organizational justice [13]. The relationship between organizational justice [13] and worker productivity [14] has all been the widest and longest research tradition [6]. The fair task allocation to the inspectors and the proper assignments of tasks regarding their requirements are vital to guarantee/develop a well working OHS Inspection system.

The main purpose of the current study is to obtain the most effective fair balanced task assignment among the inspectors. For this purpose, the proposed approach maintains committee-period-city assignments by maximizing inspectors' preference scores and balances the total distance traveled by each committee in the first phase. In the second phase, the obtained committee-period-city assignments are matched to the workplaces by balancing the workloads of committees regarding the number of employees in the workplaces in a city. The computations were performed on a personal computer by GAMS programming package [62] and the optimal solutions of models are obtained.

The task assignment obtained by the proposed approach and the implemented task schedule were compared. The implemented task schedules generated manually take more time, preferences of the inspectors are not considered, balancing workload is challenging while considering whole requirements of the problem concurrently. The proposed approach maintains the technical requirements of the problem and also provides social and physical balance by taking into account the distances covered by the work places and the number of the workplaces' employees to be audited and pays attention to the human factors by considering the preferences of the inspectors. A standard procedure to assign tasks will help management and makes inspectors' work motivation high with a fair balanced task assignment. Furthermore, the personnel who generate the schedule will be responsible to carry out properly the steps of the procedure and they will be free from the pressure of special requests.

For the planned inspections, it is recommended that the board apply the proposed approach for each project. Implementation can be facilitated by a decision support system that the proposed hierarchical solution approach (Fig. 1) is embedded into it.

Although each inspector has to fulfill certain level of job competence, they may be assigning tasks based on their technical and nontechnical skill levels for high-risk level projects in addition to the main concerns of the current study. Task assignment based on human skills [63] will be evaluated as a future research.

The proposed hierarchical models can be easily revised based on different countries' local requirements. The findings of the study offer fruitful inspirations in the area of safety management and policy.

This study contributes to the literature by the facts that the OHS inspectors' task assignment problem has not addressed previously in literature and the solution approach is novel. The contribution goes further by considering not only the technical requirements of the problem but also psychosocial factors that inspectors affected.

Fair and balanced task assignment and consideration of inspectors' nonconflicting preferences with the both legal and ethical requirements of the problem increase the moral and motivation of the inspectors and their work engagement, well-being at work, and work satisfaction. Literature shows that these psychological factors improve the employees' work quality. Therefore, the current study expects that inspectors' work productivity is affected in a positive manner and results in improvement in the qualitative and quantitative outputs of the inspection system. Surely, the performance of the overall system is affected by the performance of each single component. The improvement prospect of overall OHS system requires a longitudinal research design and it can be evaluated as a fruitful future direction away from the current study of which main purpose is to obtain a fair balanced task assignment to maintain job satisfaction and well-being at work for OHS inspectors. To increase the productivity of OHS system, whole system components should be better improved. Service modularity concept [64] may be an inspiration for enabling value creation in OHS system. According to Pekkarinen and Ulkuniemi [65], modular services are designed on service, process, and organizational levels.

To check the strengths, weaknesses, opportunities, and threats of the proposed approach, strengths, weaknesses, opportunities, and threats (SWOT) analysis [66] may be performed as a future direction.

More quantitative and qualitative research identifying the work environment of OSH inspectors and the support they need in their work require further research attention. Specifically, the following further research areas are still open in the literature:

Although the behavioral science literature indicates that managing psychosocial risks leads to improve the productivity and quality of work [67], and though fairness has been investigated in scheduling, it is still an open area to investigate psychosocial risks that OHS inspectors be exposed.

Job satisfaction or employee satisfaction is one of the most comprehensively measured and researched topics in the fields of management and organizational psychology [68]. From this point of view, researching the improvement in OHS inspectors' work performance will be another future direction.

## Conflicts of interest

The authors declare that they have no known competing financial interests or personal relationships that could have appeared to influence the work reported in this paper.
